# Intervention research in health promotion: transferability issues, from project to structuring

**DOI:** 10.1093/eurpub/ckac129.075

**Published:** 2022-10-25

**Authors:** F Cousson-Gélie

**Affiliations:** Psychology Department, University of Montpellier, Montpellier, France

## Abstract

The work carried out in intervention research has revealed the complexity of health interventions, particularly in health promotion. Even if these interventions are in themselves complex systems interacting with their context or ‘intervention systems’, PHIR is making considerable progress in understanding the mechanisms thus revealed, a key element for their transferability, which is an essential issue in public health.Transferability assesses the extent to which the outcome of a successful intervention, evaluated in one context, can be achieved in another context. It is through the concrete example of the interventional research that has made it possible to evaluate the transferability of the project ‘P2P, peer action for the prevention of smoking among high school students in vocational training”, that the issues presented in the two previous presentations will be analyzed. Using the example of this transferability study, which aimed to assess whether the P2P programme developed and conducted in the south of France is transferable to other regions and under what conditions, participants will be invited to analyse the conditions of this implementation. Thus, the question of the reproducibility of the effectiveness results with a similar population but in a different geographical context, implying differences in the functioning of the intervening structures and the high schools involved, but also in the characteristics of the high school students targeted, will be examined. After the presentation of the project and its transferability in different spaces, an interactive debate will be organised on the challenges of transferability.

